# Case Report: Prenatal diagnosis of congenital knee dislocation caused by BICD2 gene variant

**DOI:** 10.3389/fgene.2026.1938741

**Published:** 2026-10-14

**Authors:** Xiaohua Ge, Wencong Li, Hong Wei, Meijuan Liu

**Affiliations:** 1 School of Medical Imaging, Shandong Medical And Pharmaceutical University, Yantai, Shandong, China; 2 Department of Ultrasound Medicine, The Affiliated Yantai Yuhuangding Hospital of Qingdao University, Yantai, Shandong, China

**Keywords:** BICD2 gene, congenital knee dislocation, exome sequencing, micrognathia, prenatal ultrasound

## Abstract

Congenital dislocation of the knee (CDK) is a rare malformation that severely affects the quality of life of affected fetuses and imposes a heavy burden on families and society. Its etiology remains unclear and may be related to genetic factors, quadriceps atrophy, muscular nutritional imbalance, and intrauterine factors. Here we report a case of CDK diagnosed prenatally by ultrasound in a 34-year-old pregnant woman at 25 weeks of gestation. Prenatal ultrasonography revealed knee hyperextension, abnormal hand morphology, and micrognathia. Exome sequencing identified a heterozygous mutation c.1613A>G (p.Tyr538Cys) in the BICD2 gene in the fetus, which was classified as likely pathogenic (PS2+PM2) according to American College of Medical Genetics and Genomics (ACMG) criteria. This variant is associated with autosomal dominant spinal muscular atrophy with lower extremity predominance type 2 (SMALED2). The prenatal ultrasound diagnosis was confirmed by autopsy after termination of pregnancy. This case expands the prenatal phenotypic spectrum of BICD2-related disorders by detailing the ultrasound features of CDK, highlights the diagnostic value of exome sequencing when fetal anomalies are detected prenatally, and is of great significance for the diagnosis of fetal congenital anomalies and the formulation of clinical management plans.

## Introduction

A 34-year-old primigravida (G1P0) conceived by artificial insemination, with no underlying diseases or family history of genetic disorders. Nuchal translucency at 12 weeks 3 days was normal, and second-trimester screening at 17 weeks showed a borderline risk for trisomy 21 (1/790). At 25 weeks of gestation, ultrasound revealed polyhydramnios (amniotic fluid index 20.1 cm) and no fetal movements were observed. The fetal lower lip and chin lacked the normal S-shaped curve, with loss of the reverse-S sign and a mandibular facial angle of 43°(Figure 1a) (In the midsagittal plane, measure the angle between the chin–upper lip line and the nasion vertical line,the diagnostic threshold is less than 50°). The upper and lower limbs were fixed in abnormal postures: upper arms flexed across the chest with clenched fists, and lower legs crossed with both knees hyperextended (Figures 1b,c).

**FIGURE 1:**
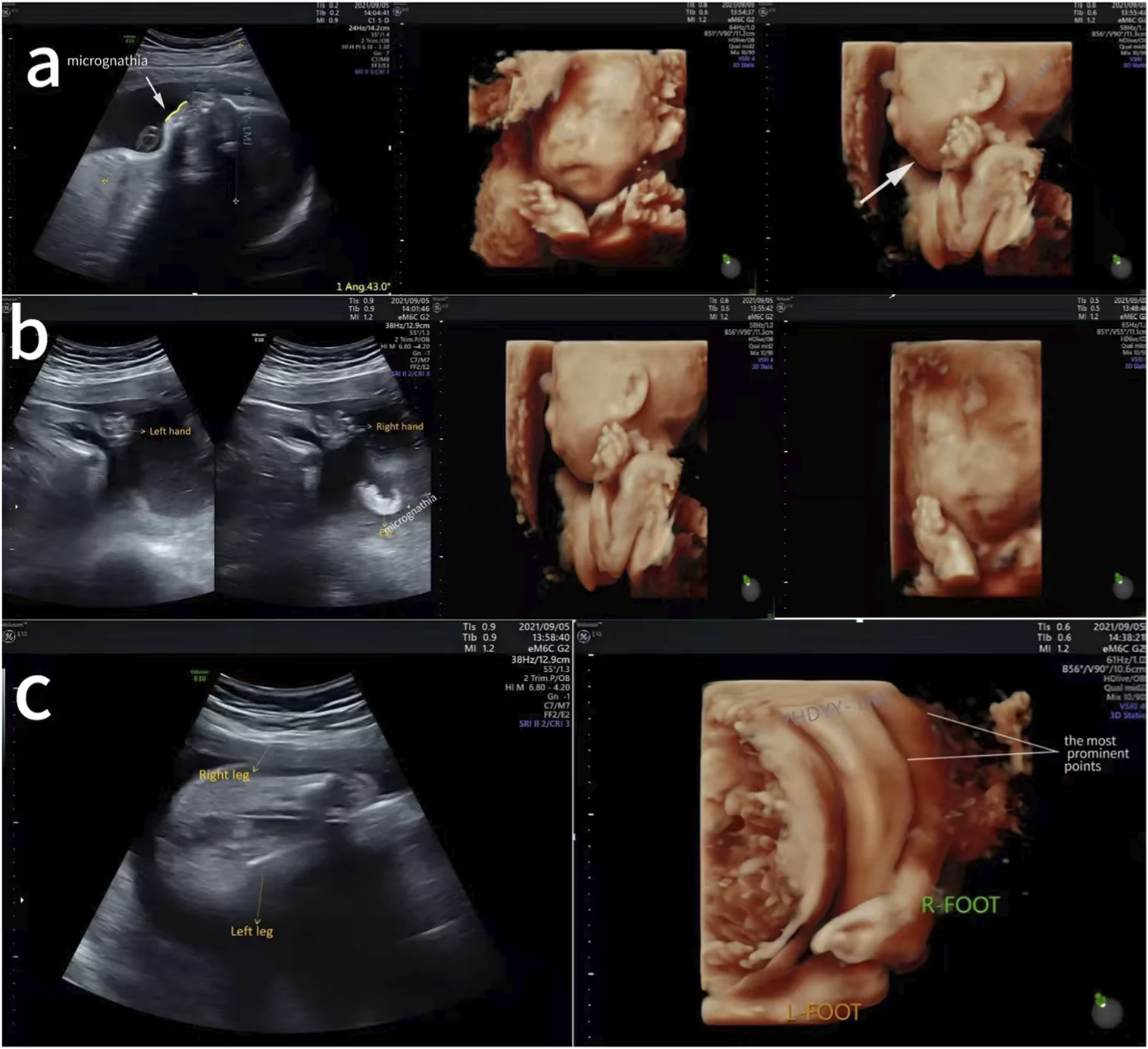
All images were obtained via transabdominal ultrasound. **(a)** Sagittal view of the fetal face: two-dimensional gray-scale ultrasound shows loss of the normal reverse-S contour of the lower lip and chin, with a mandibular facial angle of 43° (normal ≥50°); three-dimensional ultrasound with surface-rendering mode demonstrates micrognathia. **(b)** Coronal view of the fetal hands: both two-dimensional and three-dimensional ultrasound with surface-rendering mode show fixed clenched fists with persistent flexed postures. **(c)** Longitudinal view of the fetal lower limbs: two-dimensional ultrasound reveals persistently crossed and adducted lower extremities; three-dimensional ultrasound with surface-rendering mode shows that the most prominent points of both knee joints are located on the same side as the heels, indicating knee hyperextension.

After genetic counselling, whole-exome sequencing (WES) was performed on fetal cord blood, identifying a heterozygous missense variant in the BICD2 gene (NM_001003800.2:c.1613A>G, p.Tyr538Cys), which was absent in both parents. The ACMG classification was PS2+PM2 (likely pathogenic) (Figure 2; Table 1).

**FIGURE 2:**
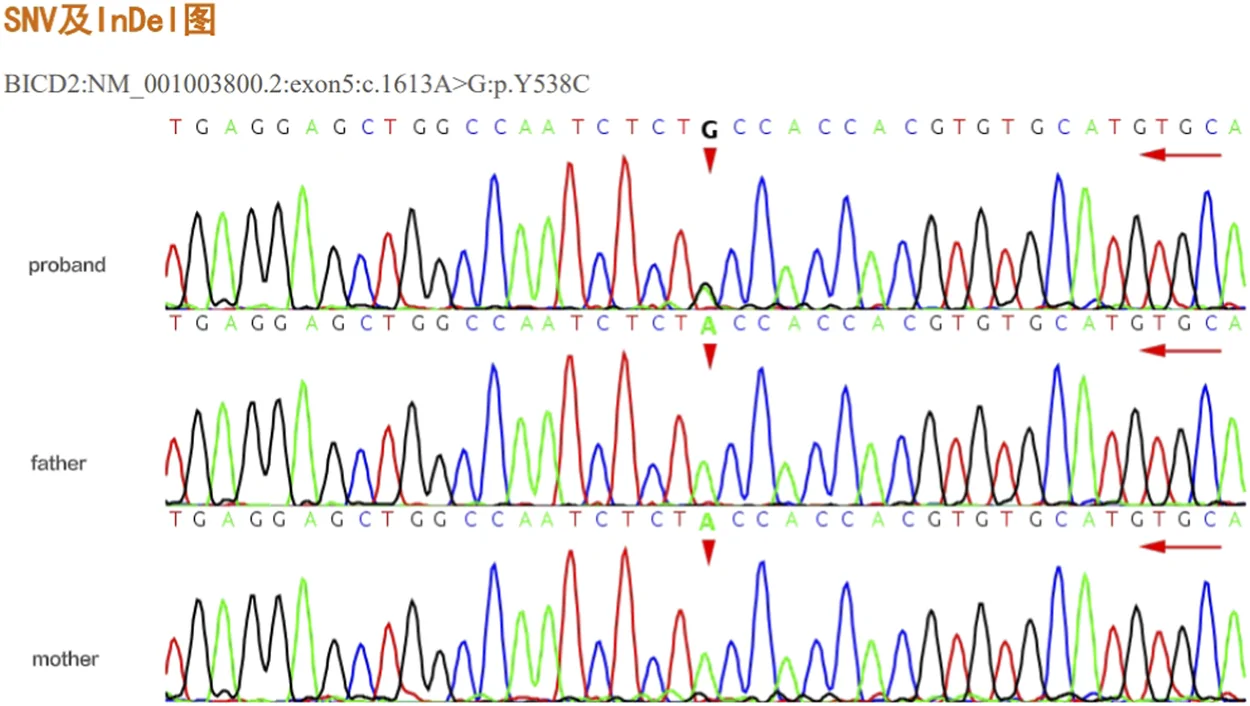
SNV and InDel results showing that the proband carried a heterozygous c.1613A>G mutation in the BICD2 gene, while the father and mother did not carry this mutation.

**TABLE 1:** Results of SNV and InDel detection.

Gene	Mutation position	Gene region	HGVS	Mutation type	Heterozygosity	Variant classification	Disease and inheritance pattern
BICD2	chr9:92,719,032-92719032	exon5	NM_001003800.2: c.1613A>G; p.Y538C	Nonsynonymous SNV	Proband: Heterozygous; father: wild type; mother: wild type	Likely pathogenic	Spinal muscular atrophy, lower extremity-Predominant, type 2A, AD; spinal muscular atrophy, lower extremity-Predominant, type 2B, AD

Following multidisciplinary discussion, the woman opted for termination of pregnancy at 25 weeks via intra-amniotic instillation of ethacridine lactate. Post-termination external examination and X-ray confirmed bilateral upper-limb flexion contractures, thumbs in palms, other four fingers clenched, crossed lower limbs, knee hyperextension, and micrognathia (Figures 3A,B); autopsy findings were consistent with prenatal ultrasound and genetic diagnosis.

**FIGURE 3:**
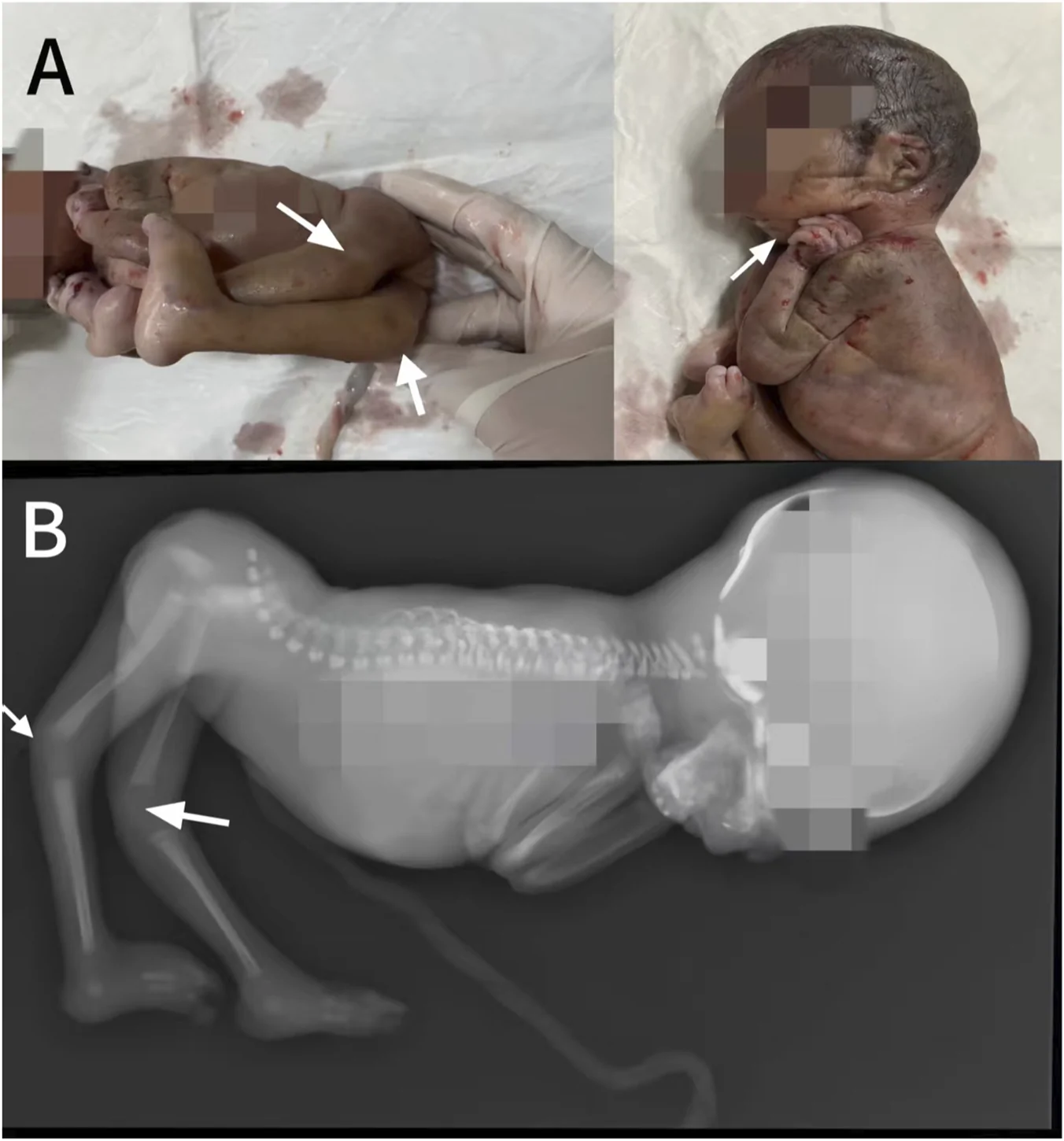
**(A)** Post-mortem gross photographs confirming micrognathia, fixed upper limbs, clenched fists, and bilateral genu recurvatum. **(B)** Post-mortem X-ray confirming fixed postures and bilateral genu recurvatum.

## Discussion

BICD2 is a dynein-1 cargo adaptor that recruits cytoplasmic dynein to diverse forms of subcellular cargo for their intracellular transport. It can recruit dynein to RNA, viral particles, and nuclei. As a cargo adaptor, BICD2 is crucial for coupling motor proteins with their respective cargoes and regulatory proteins (Fagiewicz et al., 2022; Yi et al., 2023). BICD2 contains three major coiled-coil segments. Dynein and dynactin bind to CC1, kinesin binds to CC2, and known cargoes interact with the C-terminal CC3 (Vallee et al., 2020).

Mutations in the BICD2 gene disrupt dynein-mediated transport, impair motor neuron function, and particularly affect lower-limb motor neurons. BICD2 is the causative gene for autosomal dominant spinal muscular atrophy, lower extremity dominant 2 (SMALED2). The genotype–phenotype correlation for BICD2-related SMALED2 is relatively clear: *de novo* mutations are usually associated with a more severe phenotype, and variants in the CC2 region and the C-terminal cargo-binding region more often cause severe disease. The CC2 region variant p.Arg501Pro is associated with SMALED2 (Koboldt et al., 2020; Oates et al., 2013).

In this case, p.Tyr538Cys is located in the CC2 domain. The CC2 domain mediates the binding of BICD2 to KIF5B and participates in the coordination of bidirectional transport. The specific functional consequences of this variant have not yet been experimentally verified. However, given its localization in the CC2 domain, the precedent of pathogenic variants in the same region such as p.Arg501Pro, and the severe prenatal phenotype of the fetus in this case—including fixed lower-limb posture, bilateral knee hyperextension, multiple joint contractures, micrognathia, and polyhydramnios—this variant is consistent with the genotype–phenotype association of BICD2-related SMALED2. This suggests that when the above signs are detected prenatally, BICD2-related SMALED2 should be considered and trio-WES should be performed as early as possible.

Prenatal ultrasound diagnosis of congenital knee hyperextension (CDK) uses sequential scanning along the long axis of the limbs to detect structural anomalies.CDK may be isolated or syndromic. Differential diagnosis includes congenital myopathies (RYR1, NEB, ACTA1; autosomal recessive/dominant), especially RYR1/NEB-related, which prenatally show hydrops fetalis, large cystic hygromas, and bilateral talipes (Jackson et al., 2024; Pérez-Vidarte et al., 2025). Larsen syndrome (FLNB-related; autosomal recessive/dominant) prenatally shows multiple joint dislocations/anterior knee dislocation, spinal anomalies, and multisystem abnormalities (Siafaka et al., 2023); BICD2-SMALED2 lacks these and is autosomal dominant.As for treatment, isolated CDK is treated conservatively with casting, orthoses, and gait training; surgery is reserved for refractory cases and prognosis is favourable, whereas syndromic CDK has poorer outcome and often requires surgery (Qi et al., 2024).

In summary, this case reports a prenatal diagnosis of CDK caused by a *de novo* BICD2 variant (p.Tyr538Cys). When fetal micrognathia and lower-limb postural abnormalities are detected, BICD2-related SMALED2 should be considered in the differential, and trio-WES should be performed early to guide genetic counselling and clinical management.

## Data Availability

The datasets presented in this study can be found in online repositories. The names of the repository/repositories and accession number(s) can be found in the article/supplementary material.
